# Sequential multiplex PCR assay for determining capsular serotypes of colonizing *S. pneumoniae*

**DOI:** 10.1186/1471-2334-11-100

**Published:** 2011-04-20

**Authors:** Sarah Jourdain, Pierre-Alexandre Drèze, Jozef Vandeven, Jan Verhaegen, Laurence Van Melderen, Pierre R Smeesters

**Affiliations:** 1 Laboratoire de Génétique et Physiologie Bactérienne, Institut de Biologie et de Médecine Moléculaires, Université Libre de Bruxelles, Bruxelles, Belgium; 2 Paediatric Department, Hôpital Universitaire des Enfants Reine Fabiola HUDERF, Université Libre de Bruxelles, Bruxelles, Belgium; 3 Pneumococcus Reference Laboratory, Katholieke Universiteit Leuven, Leuven, Belgium

## Abstract

**Background:**

Asymptomatic nasopharyngeal carriage represents an important biological marker for monitoring pneumococcal serotype distribution and evaluating vaccine effects. Serotype determination by conventional method (Quellung reaction) is technically and financially challenging. On the contrary, PCR-based serotyping represents a simple, economic and promising alternative method.

**Method:**

We designed a novel multiplex PCR assay for specific detection of the 30 classical colonizing *S. pneumoniae *serogroups/types. This multiplex assay is composed of 7 consecutive PCR reactions and was validated on a large and recent collection of *Streptococcus pneumoniae *isolated during a prospective study conducted in Belgium at the time of PCV7 adoption.

**Results:**

The multiplex PCR assay allowed the typing of more than 94% of the isolates of a collection of pneumococci isolated from Belgian preschool attendees (n = 332). Seventy-five percent of the isolates were typed after 3 subsequent PCR reactions. Results were in agreement with the Quellung identification.

**Conclusion:**

Our novel multiplex assay is an accurate and reliable method which can be used in place of the conventional method for *S. pneumoniae *carriage studies.

## Background

*S. pneumoniae *is a major cause of morbidity and mortality worldwide. The World Health Organisation estimates that 1.6 million people among which 0.7 to 1 million children under 5 die of pneumococcal diseases each year [1]. The nasopharynx of young children represents the ecological niche of *S. pneumoniae *as well as the reservoir for pneumococcal transmission within the community. Colonisation is also recognized as the first step of disease development [2]. *S. pneumoniae *major virulence factor is the capsular polysaccharide upon which *S. pneumoniae *classification is based. Today, 93 different serotypes (including the recently discovered 6D and 11E [3,4]), belonging to 46 serogroups, have been described [5,6]. Serotypes differ by geographical prevalence [7], attack rate [8], age distribution [7] tendency to cause outbreaks [9,10] and propensity to acquire antimicrobial resistance genes [11-13]. The capsular polysaccharide is also the target of pneumococcal vaccine. The currently available protein conjugate vaccine, PCV7, protects against 7 clinically-relevant serotypes in young children (4, 6B, 9V, 14, 18C, 19F, 23F). Because of its coverage limitation, it has induced replacement of vaccine serotypes strains (VT) by non-vaccine serotypes strains (NVT) in colonisation and subsequently in disease [14,15]. The long-term effects of a vaccine targeting only a restricted number of serotypes remain unknown. It is therefore crucial to keep ongoing surveillance of pneumococcal serotypes in invasive diseases [15,16] and in carriage [17-19].

Serotype determination relies on the Quellung reaction. Binding of specific monoclonal antibodies with the capsule induces a capsular swelling visualised under the microscope. This technique remains the gold standard for pneumococcal serotyping but presents major drawbacks: high cost of antisera, technical expertise requirements and subjectivity in interpretation of results [20]. All these hindrances restrict *S. pneumoniae *serotyping to a few reference laboratories [21].

The capsular polysaccharides synthesis pathway is encoded by the *cps *(capsular polysaccharide synthesis) locus. Sequencing of the *cps *locus of the 93 known serotypes provided the molecular basis for PCR-based serotyping developments [22]. A first sequential multiplex PCR assay specific to invasive pneumococcal diseases (IPD) distribution in the USA was developed by Pai and colleagues [23]. This initial assay was modified based on IPD epidemiology encountered in different geographical areas (Brazil, Mozambique and Spain) [21,24,25]. It was also successfully applied for *S. pneumoniae *serotypes detection from nasopharyngeal (colonizing) and cerebro-spinal fluid (IPD) samples [21,26-28] (Table 1). To conclude, most of the multiplex PCR assays developed so far are specific to IPD strains. Only two studies (in the USA and the Gambia) have used the IPD designed multiplex PCR in carriage studies [28,29]. To optimize the rapid detection of carriage-associated serotypes, we developed a sequential multiplex PCR assay specific to colonizing pneumococci. This assay was experimentally tested on a collection of more than 300 pneumococci isolated in Belgium and representing carriage serotypes distribution in Europe. This new multiplex scheme successfully identified 95% of the isolates.

**Table 1:** Utilization and adaptation of the original multiplex published by Pai and colleagues

	Year	Country	Age	Aim of the study	Isolates	Method	No of samples	Concordance *	Nb serotypes/groups	Ref
**Plating**										
	2006	USA	All	develop and validate the multiplex	IPD (blood)	multiplex PCR	421	100%	29	[23]
	2007	Mozambique	< 15 years	adapted to African epidemiology	IPD (blood+CSF)/AOM	multiplex PCR	153	91.50%	29	[21]
	2007	Brazil	< 5 years	adapted to Latin American epidemiology	IPD (blood+CSF)/pleural fluid	multiplex PCR	147	94.60%	30	[25]
	2010	Spain	All	adapted to European epidemiology	IPD	multiplex PCR	257	95.7%	29	[24]
**No plating**										
	2008	Italy	0-14 years	PCR-based serotyping directly on clinical specimens	IPD (blood+CSF)/pleural fluid	RT-PCR + multiplex PCR	92	NA	31	[35]
	2009	Gambia	All	detect multiple carriage	carriage	multiplex PCR	279	NA	29	[29]
	2010	USA	< 2 years	detect multiple carriage+low density	carriage	Broth enrichment + RT-PCR + multiplex PCR	100	NA	40	[28]
	2008	Bangladesh	ND	PCR-based serotyping directly on clinical specimens	meningitis (CSF+isolates)	multiplex PCR on isolates and directly on CSF	358	NA	56	[26]
	2010	Burkina Faso and Togo	All	PCR-based serotyping directly on clinical specimens	meningitis (CSF+isolates)	multiplex PCR on isolates and directly on CSF	194	NA	29	[27]

* between conventional and PCR-based serotyping. IPD Invasive Pneumococcal Disease; CSF Cerebrospinal Fluid; AOM Acute Otitis Media; ND Not Determined; NA Not applicable (Studies use PCR to yield additional results compared to bacterial isolation and conventional serotyping, no concordance can be calculate

## Methods

### Study design

We carried out a prospective cohort study on healthy children (3-6 year-old) from 11 preschools of the Brussels area between September 2006 and May 2008, time of PCV7 introduction in Belgium [30]. Nasopharyngeal aspirates were performed three times during the school years on 333 children (mean age 4.2 years). A total of 830 samples were analysed for the presence of *S. pneumoniae *and 3 other potential pathogens (*S. aureus, H. influenzae *and *M. catarrhalis*). Sixty-nine percent of the children carried at least once *S. pneumonia*e. Number of colonies per sample was not determined. Only one colony of each bacterial species was included per sample.

Detailed information about the study was provided to parents and school staffs. The Ethics Committee of the Hôpital Universitaire des Enfants Reine Fabiola approved the study. Signed informed consents were obtained from parents.

### Bacterial strains

A collection of 362 *S. pneumoniae *strains were isolated from healthy children [30]. *S. pneumoniae *isolates were identified by optochine-susceptibility test, bile solubility test and a positive *lytA *PCR as described previously [31].

### *S. pneumoniae *typing

*S. pneumoniae *isolates were serotyped by Quellung reaction performed by 2 different experimenters. Thirty-three different serogroups/types were identified [30]. Thirty isolates were assigned as non-typeable pneumococci (NTP). These 30 isolates were excluded from this study. The vast majority of these isolates did not show any amplification for the *cpsA *internal positive control (data not shown). A total of 332 isolates was therefore included in this study. Serotypes of these isolates were also determined using the original multiplex PCR assay described in Pai and colleagues [23]. Additional primers described in [25,26] as well as primers specific to serotype 6C [32], 23A, 23B and 35A (newly designed by the CDC, (http://www.cdc.gov/ncidod/biotech/strep/pcr.htm)) were tested. All the primers used in this study have therefore been previously published (Table 2). Primers specific to the *cpsA *gene were used as an internal positive control. This gene is part of the *cps *locus and is highly conserved in pneumococci [23].

**Table 2:** Multiplex primer

Name	Sequences (5' → 3')	Amplicon size
1-f	CTC TAT AGA ATG GAG TAT ATA AAC TAT GGT TA	280
1-r	CCA AAG AAA ATA CTA ACA TTA TCA CAA TAT TGG C	
3-f	ATG GTG TGA TTT CTC CTA GAT TGG AAA GTA G	371
3-r	CTT CTC CAA TTG CTT ACC AAG TGC AAT AAC G	
4-f	CTG TTA CTT GTT CTG GAC TCT CGA TAA TTG G	430
4-r	GCC CAC TCC TGT TAA AAT CCT ACC CGC ATT G	
6-f	AAT TTG TAT TTT ATT CAT GCC TAT ATC TGG	250
6-f	TTA GCG GAG ATA ATT TAA AAT GAT GAC TA	
6C-f	CAT TTT AGT GAA GTT GGC GGT GGA GTT	727
6C-r	AGC TTC GAA GCC CAT ACT CTT CAA TTA	
7C/B (40)-f	CTA TCT CAG TCA TCT ATT GTT AAA GTT TAC GAC GGG A	260
7C/B (40)-r	GAA CAT AGA TGT TGA GAC ATC TTT TGT AAT TTC	
7A/F-f	CCT ACG GGA GGA TAT AAA ATT ATT TTT GAG	826
7A/F-r	CAA ATA CAC CAC TAT AGG CTG TTG AGA CTA AC	
9A/V-f	CTT CGT TAG TTA AAA TTC TAA ATT TTT CTA AG	753
9A/V-r	GTC CCA ATA CCA GTC CTT GCA ACA CAA G	
10A-f	GGT GTA GAT TTA CCA TTA GTG TCG GCA GAC	628
10A-r	GAA TTT CTT CTT TAA GAT TCG GAT ATT TCT C	
11A/D/F-f	GGA CAT GTT CAG GTG ATT TCC CAA TAT AGT G	463
11A/D/F-r	GAT TAT GAG TGT AAT TTA TTC CAA CTT CTC CC	
12F (12A/B, 44, 46)-f	GCA ACA AAC GGC GTG AAA GTA GTT G	376
12F (12A/B, 44, 46)-r	CAA GAT GAA TAT CAC TAC CAA TAA CAA AAC	
14-f*	GAA ATG TTA CTT GGC GCA GGT GTC AGA ATT	189
14-r*	GCC AAT ACT TCT TAG TCT CTC AGA TGA AT	
15A/F-f	ATT AGT ACA GCT GCT GGA ATA TCT CTT C	434
15A/F-r	GAT CTA GTG AAC GTA CTA TTC CAA AC	
15B/C-f	TTG GAA TTT TTT AAT TAG TGG CTT ACC TA	496
15B/C-r	CAT CCG CTT ATT AAT TGA AGT AAT CTG AAC C	
16F-f	CTG TTC AGA TAG GCC ATT TAC AGC TTT AAA TC	988
16F-r	CAT TCC TTT TGT ATA TAG TGC TAG TTC ATC C	
17F-f	TTC GTG ATG ATA ATT CCA ATG ATC AAA CAA GAG	693
17F-r	GAT GTA ACA AAT TTG TAG CGA CTA AGG TCT GC	
18-f	CTT AAT AGC TCT CAT TAT TCT TTT TTT AAG CC	573
18-r	TTA TCT GTA AAC CAT ATC AGC ATC TGA AAC	
19A-f	GTT AGT CCT GTT TTA GAT TTA TTT GGT GTT GT	478
19A-r	GAG CAG TCA ATA AGA TGA GAC GAT TGT TAG	
19F-f	GTT AAG ATT GCT GAT CGA TTA ATT GAT ATC C	304
19F-r	GTA ATA TGT CTT TAG GGC GTT TAT GGC GAT AG	
22-f	GAG TAT AGC CAG ATT ATG GCA GTT TTA TTG TC	643
22-r	CTC CAG CAC TTG CGC TGG AAA CAA CAG ACA AC	
23A-f***	TAT TCT AGC AAG TGA CGA AGA TGC G	722
23A-r***	CCA ACA TGC TTA AAA ACG CTG CTT TAC	
23B-f**	TTG TTA GTG GTA TTA AAT TGG GGA CTA CTA GG	216
23B-r**	ATA CCT ATC TGA AGT GTT ATT AAC CCA CCA AC	
23F-f	GTA ACA GTT GCT GTA GAG GGA ATT GGC TTT TC	384
23F-r	CAC AAC ACC TAA CAC TCG ATG GCT ATA TGA TTC	
24A/F-f**	TCT CAA CCA AGA TAC AGA TTT TGA TTT TAC TC	686
24A/F-r**	TAT AAA CCT TTA GTA AAC ACT CTG CTT GAT CG	
31-f	GGA AGT TTT CAA GGA TAT GAT AGT GGT GGT GC	701
31-r	CCG AAT AAT ATA TTC AAT ATA TTC CTA CTC	
33F (33A/37)-f	GAA GGC AAT CAA TGT GAT TGT GTC GCG	338
33F (33A/37)-r	CTT CAA AAT GAA GAT TAT AGT ACC CTT CTA C	
34-f	GCT TTT GTA AGA GGA GAT TAT TTT CAC CCA AC	408
34-r	CAA TCC GAC TAA GTC TTC AGT AAA AAA CTT TAC	
35A (35C/42)-f***	ATT ACG ACT CCT TAT GTG ACG CGC ATA	280
35A (35C/42)-r***	CCA ATC CCA AGA TAT ATG CAA CTA GGT T	
35B-f	GAT AAG TCT GTT GTG GAG ACT TAA AAA GAA TG	677
35B-r	CTT TCC AGA TAA TTA CAG GTA TTC CTG AAG CAA G	
35F (47F)-f	GAA CAT AGT CGC TAT TGT ATT TTA TTT AAA GCA A	517
35F (47F)-r	GAC TAG GAG CAT TAT TCC TAG AGC GAG TAA ACC	
38 (25A/F)-f	CGT TCT TTT ATC TCA CTG TAT AGT ATC TTT ATG	574
38 (25A/F)-r	ATG TTT GAA TTA AAG CTA ACG TAA CAA TCC	
cpsA-f	GCA GTA CAG CAG TTT GTT GGA CTG ACC	160
cpsA-r	GAA TAT TTT CAT TAT CAG TCC CAG TC	

The majority of primer sequences are available on the CDC website (http://www.cdc.gov/ncidod/biotech/files/pcr-oligonucleotide-primers-March2010.pdf); *, ** and *** indicate primer sequences published by [25,26], CDC website respectively. The name of each primer has been modified accordingly to the results obtained with the primer blast software.

In case of non-concordant results, serotypes were double-checked using the Quellung reaction and the multiplex PCR assay. In case of non-concordance, the national reference laboratory confirmed the Quellung identification.

Molecular methods as DNA extraction, PCR and PCR product detection on 2% agarose gel were performed as described in [23]. Briefly, a bacterial suspension with a turbidity of MacFarland standard 1 was boiled at 100°C for 5 min and frozen at -20°C for 5 min. The PCR reactions were performed in 25 μl with 2.0 U of *taq *DNA polymerase (Promega Inc.), 5 μl of Promega green buffer 5X, 2.5 mM magnesium chloride, 200 μM of each DNTP (Promega Inc.), 2.5 μl of bacterial crude extract and primers with concentration as specified in Table 3. PCR reactions were performed using a Biometra T3000 thermocycler using the following conditions: 4 min at 94°C followed by 30 cycles composed of 45s at 94°C, 45s at 54°C and 150s at 65°C.

**Table 3:** Multiplex design

Primers		Primer concentration (μM)
Reaction 1	6A/B/C	1
	23F	1.5
	11A	1.5
	23A	2
Reaction 2	23B	0.75
	19F	1
	19A	1.5
	38	1.5
Reaction 3	1	0.75
	3	1
	15B/C	1.25
	35B	1.5
Reaction 4	14	0.75
	34	0.75
	22F	1.25
	9A/V	1.5
Reaction 5	35A	0.75
	12F	1
	24A/F	1.25
	16F	1.25
	7A/F	2
Reaction 6	33F	1
	4	1
	35F	1.25
	10A	1.5
	31	1.5
Reaction 7	7B/C	0.75
	15A	1
	18C	1
	17F	1.25

Primers *cpsA*-f and *cpsA*-r were included in all reactions at 0.5 μM each

## Results

### In silico design of the multiplex PCR assay

A novel multiplex PCR assay was developed for specific detection of the 30 classical colonizing *S. pneumoniae *serogroups/types (Table 3). This multiplex assay is composed of 7 consecutive PCR reactions combining primers that have been already described in different studies [23,25,26] (Table 3). Serotypes included in the 7 PCR reactions were chosen upon their prevalence in carriage studies [7] to maximize serotypes determination in a limited number of PCR reactions. Primers were combined in each reaction to yield differences of more than 70 bp in PCR fragment size for non-ambiguous interpretation. Each PCR reaction includes a primer pair specific to the *cpsA *gene as internal positive control.

The specificity of the selected primer pairs was assessed *in silico *with the Primer-Blast software (http://www.ncbi.nlm.nih.gov/tools/primer-blast/index.cgi?LINK_LOC=BlastHome) (default parameters with 'nr' database and '*Streptococcus pneumoniae *taxid' species). Table 2 shows that 18 primer pairs are predicted to be specific to a given serotype (10A, 16F, 17F, 19A, 19F, 23A, 23B, 23F and 35B) or serogroup (1, 3, 4, 6, 14, 18, 22, 31 and 34) while the 12 others might present some cross-reactivity between different serotypes (7A/F, 9A/V, 11A/D/F, 15A/F, 15B/C and 24A/F) or serogroup (7C/B with 40; 12F with 12A/B, 44 and 46; 33F with 33A and 37; 35A with 35C and 42; 35F with 47F; 38 with 25A/F). Some serotypes are only occasionally associated with carriage and were therefore not included in the multiplex (e.g. serotypes 2, 5, 28, 29 and 46) [7].

### Experimental validation of the multiplex PCR assay

**Figure 1:**
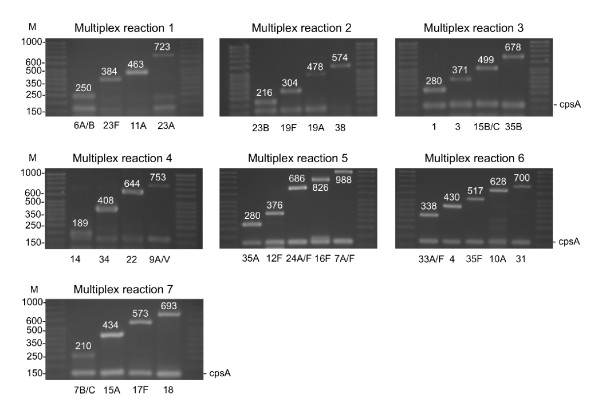
**Multiplex PCR reactions adapted to colonizing pneumococci**. M means molecular size in bp.

The novel multiplex PCR assay was experimentally validated on a large and recent collection of *S. pneumoniae *isolated from Belgian preschool healthy children at the time of PCV7 adoption [30]. This collection included 332 typable isolates belonging to 31 serogroups/types assigned by Quellung reaction. Three serotypes (13, 28, 29) were not included in our PCR scheme. The PCR discriminates serotype 15A from serotypes 15B/C and serotype 35F from 35A. In total, 329 of the isolates (96%) belong to the 30 serotypes included in the PCR scheme. The 7 PCR reactions were tested on every isolate of this collection. During the experimental validation, we did not observe any primer inhibition and specific identification of each serotype was accurate (Figure 1).

**Figure 2:**
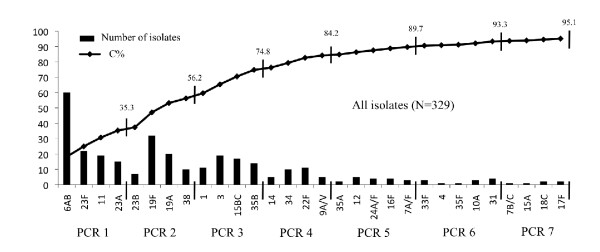
**Serotype coverage of the Multiplex PCR**. Number of pneumococcal isolates and cumulative frequencies for each serotype. C%, Cumulative Frequencies.

The first PCR reaction (PCR-1) includes primers specific to serogroup/type 6, 23F, 11A and 23A. About one third of our collection was serotyped after PCR-1 (116/329 isolates, 35.25%) (Figure 2). Serogroup 6 primers cannot discriminate 6A and 6B isolates because of the high sequence similarity in the target gene (*wciP*). On the other hand, the newly discovered and emerging serotype 6C can be identified by PCR. We additionally tested the 60 serogroup 6 isolates using 6C primers (Primers described in Table 2, PCR condition as described in material and methods). Serotype 6C is recognized as an emerging serotype following PCV7 adoption [33,34] and represents currently the predominant serogroup 6 serotype recovered in diseases in the USA [32]. No 6C isolates were present in our collection. The amplification of the internal control (*cpsA*) was present for 115 of the 116 strains identified by PCR-1. *cpsA *control amplification was missing for one 23A isolate (only the 23A specific amplicon of 723 bp was present). Twenty-two serotype 23A and 29 serotype 23F pneumococci are present in our collection. PCR-1 successfully identified 15 (68%) 23A and 22 (76%) 23F but no specific amplification was observed for 7 serotype 23A (32%) and 7 serotype 23F (24%). Subsequent testing of these strains with specific antisera at the National reference laboratory for pneumococci confirmed serotype 23A and 23F identification. The PCR identified accurately all other serotypes included in PCR-1 (6 and 11A). In total, PCR-1 successfully identified 116 (88%) of the 130 isolates belonging to serotypes 6, 23F, 11A and 23A. The experimental validation of the 4 primer pairs included in PCR-1 was 100% specific as no false positive results were obtained and no discrepancies with Quellung identification were observed. PCR-1 identified 37 serogroup 23 pneumococci but could not assign a serotype to 14 serogroup 23 strains.

The second PCR reaction (PCR-2) is specific to serotypes 23B, 19F, 19A and 38. This reaction assigned a serotype to 69 isolates. After the 2 first reactions, more than half of the isolates were typed (185/329; 56.23%). *cpsA *internal control was missing for 9 out of 10 serotype 38 isolates but was present in every other isolate. In comparison to the Quellung serotyping, PCR-2 presented 97% sensitivity and 100% specificity. Only 2 out of 9 serotype 23B pneumococci (confirmed by the National reference laboratory) did not present any specific amplification.

The third reaction, PCR-3, detects serotypes 1, 3, 15B/C and 35B. This reaction allowed for serotype identification of 61 isolates. After 3 reactions, 74.77% of our isolates were serotyped and the data were in agreement with conventional serotyping (100% sensitivity and specificity).

PCR-4 (including serotypes 14, 34, 22F, 9A/V), PCR-5 (35A, 12F, 24A/F, 16F, 7A/F), PCR-6 (33F, 4, 35F, 10A, 31) and PCR-7 (7B/C, 15A, 17F, 18) allowed for the identification of 9%, 5%, 4% and 2% of the collection respectively (cumulative frequencies presented in Figure 2). The sensitivity and specificity were 100% for the last 4 PCR reactions.

In total, PCR-deduced results were concordant with conventional serotyping and allowed for the accurate identification of 313 out of 329 pneumococci of our collection (95.13%) (Figure 2).

## Discussion

PCR schemes were developed to detect IPD associated serotypes distribution [21,23-26]. Different authors have validated, adapted and improved the original multiplex PCR assay developed by Pai and colleagues [23]. These studies are summarized in table 1. The authors demonstrated the specificity of primers for IPD isolates in Africa [21], Asia [26], South-America [25] and Europe [24]. Moreover, PCR results were in agreement with conventional serotyping (91 to 100%) and a new PCR scheme was proposed for each location. The PCR multiplex assay was also used to detect carriage isolates in two studies [28,29]. However, as serotypes associated with carriage and with diseases differ, the proposed PCR schemes were not optimal to rapidly serotype colonizing pneumococci. We thus designed a multiplex PCR scheme specific to carriage-associated serotypes. This assay can be used worldwide since serotype distribution of colonizing isolates is quite similar between different geographic locations. The most frequent serogroups are 6, 14, 19 and 23 [7]. Other colonizing serogroups frequently isolated from children are 3, 4, 9, 11, 13, 15, 18 and 33 [7].

The multiplex assay requires little technical skills and only basic PCR capabilities that are nowadays broadly accessible. Moreover, colonizing pneumococci represent a potent biological tool to monitor serotypes distribution and antimicrobial resistance level. Indeed, considering serotype prevalence in colonisation, rather than in disease, may be more accurate, faster and cheaper to evaluate pre- and post-vaccine product adoption [29]. The main disadvantage of PCR-based serotyping is the incapacity to discriminate closely-related serotypes such as 6A and 6B, 7F and 7A, 33F, 33A and 37, 35A, 35C and 42 (Table 2) but this accounts mainly for minor serotypes. Moreover, our *in silico *analysis of primer specificity showed that some primer pairs are predicted to present some cross-reactivity with other serogroups (e.g. primer pair for 7C/B, 12F, 33F, 35A, 35F and 38). This potential drawback could not be experimentally assessed in our collection as several serogroups were lacking in our collection. However, no false-positive result was obtained. As a consequence, when an amplicon of the proper size is obtained, isolates do not need to be tested with the following PCR reactions, as described previously [23].

The present scheme successfully typed 95.1% of our isolates belonging to serotypes included in the multiplex assay. This corresponds to 94.3% of the whole collection since primer pairs specific to 3 serotypes (13, 28 and 29, corresponding to three isolates) are not present in our assay. This validation confirms the flexibility of the multiplex PCR method because different primer pair combinations can be used. Moreover our method is rapid as 75% of our isolates were assigned a serotype after only 3 PCR reactions. For colonizing pneumococci, 2 studies evaluated the use of a PCR method to detect low colonisation rate and multiple colonizing serotypes from nasopharyngeal samples [28,29]. This approach of direct serotype determination, without bacterial plating and isolation, is attractive but shows limitations such as incapacity to determinate antibiotics resistance profile or to apply complementary typing tools such as MLST.

Primers accurately identified the majority of serogroups/types included in our scheme but a lack of sensitivity of primers specific to serogroup 23 was observed. The sixteen strains for which no serotype could be assigned by PCR are belonging to serogroup 23 and represent 26.7% of the total serogroup 23 pneumococci isolated in our study (16/60). The Belgian serogroup 23 colonizing pneumococci present most likely sequence variability in the target gene (*wzy *for serotype 23A and 23F, *wzx *for serotype 23B) and are therefore not amplified by the current primer pairs. This demonstrates the need to validate the PCR-based method in different populations and locations to assess primers accuracy and specificity within a given serotype. Moreover, isolates that are non-typable by PCR should be monitored with Quellung technique to assess for the emergence of variants in pneumococcal serotypes.

In our collection, several pneumococci were assigned a serotype by multiplex PCR but did not present a *cpsA*-specific amplification (3%). This was already observed by Carvalho and colleagues at a rate of 1 to 2% [28]. This particularity is serotype dependent with serotype 38 and 25F generally negative for *cpsA*. We confirm this observation for colonizing serotype 38 (9 out of 10 isolates were cpsA negative). Variation within a serotype, incapacity to discriminate serotypes and occasional absence of the internal positive control represent the major weaknesses of the PCR-based method. These problems will likely be overcome in the near future with increasing sequencing of pneumococcal capsule locus. Sequence comparisons will enable the identification of conserved and specific sequences within a given serotype as well as the detection of conserved regions through 93 serotypes to design a reliable positive control. This will undoubtedly improve the PCR-based method.

## Conclusions

To conclude, we have developed the first multiplex PCR assay designed for colonizing pneumococci. In the present study, we demonstrate that the PCR-based method is a simple, robust and economical method for serotype determination of colonizing pneumococci. We also demonstrate the accuracy of primers for carriage isolates in Belgium. This assay represents a good alternative to the Quellung reaction.

## Abbreviations

PCV7: heptavalent pneumococcal conjugate vaccine; VT: vaccine types; NVT: non vaccine types; NTP: Non typeable pneumococci; IPD: invasive pneumococcal diseases; bp: base pair; MLST: Multi Locus Sequence Typing.

## Competing interests

PRS has received a research grant from Pfizer.

JVe is member of a steering committee of a research project sponsored by Pfizer. JVe is heading the national reference laboratory for *S. pneumoniae *that received grants from Pfizer and GSK for serotyping isolates.

S J, PD, JV, LV: Nothing to declare

## Authors' contributions

SJ and PRS conceived the study and wrote the paper with LV. SJ and PD performed multiplex PCR. SJ and JVa performed pneumococci serotyping. SJ, PRS, LV and Jve analysed the data. All authors read and approved the final manuscript.

## Pre-publication history

The pre-publication history for this paper can be accessed here:

http://www.biomedcentral.com/1471-2334/11/100/prepub
